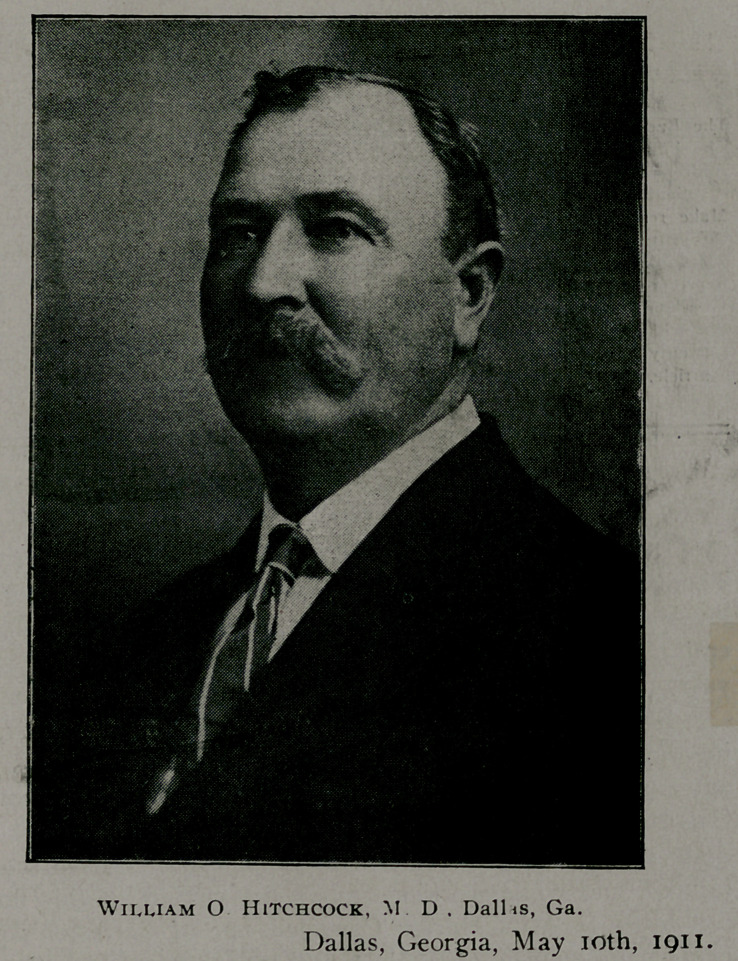# Office Hygiene

**Published:** 1911-05

**Authors:** 


### COMMUNICATIONS

### OFFICE HYGIENE.

Editor Journal-Record:—

It is quite possible that this field has been covered before, but at the same time it is well to bring up occasionally this very needful and timely thought.

Can we not often plead guilty in this particular, even the best of us? To begin with, do we always have the cleanest of offices? Are not the walls hung with pictures and bric-a-brac from one year to another without thoroughly cleansing? Do we not often hang our walls in our reception rooms with diplomas and other cut and dried objects and in the center of the table a microscope for the edification of our patients? It certainly looks as if it impresses the patient, and if they thought about it at all it would strike them as a queer ornament, and through the summer fur rugs and the same old winter carpets still on the same germ-laden floor.

Speaking of diplomas—they are all right in their place, in tan cases; do you think a patient enjoys looking at them or cheap lithographs? Let us realize this and we will all have a house cleaning which will relegate this rubbish to the dust heap, where it properly belongs. After getting our offices clean, including walls and floors, let us see that we have ventilation, not draughts. Of all the sins on the professional calendar this is the worst—bad air. The fresh air entirely excluded, and especially when there are three or four doctors occupying the offices at different hours, it makes the matter more imperative.

If windows cannot be raised or lowered, there are ventilators to be had so the air can come in and out without any draught. All hospitals and well-ventilated offices have this precaution.

The doctor then comes in for the most rigid cleanliness as regards himself, especially the smokers. It does not seem possible to me how the physician, or especially the surgeon, can possibly smoke in office hours. This habit if indulged in, should be done at home, where their comfort cannot be disturbed, and where no one objects. It seems professional men and women should strive hard for this worthy and laudable object in our offices, as men will thoughtlessly come in women's offices smoking and seem surprised when asked to lay aside the weed.

Next in order, an office coat and clean hands and smooth faces are required, and are we asking too much, when we ask, clean minds? The next step, clean instruments, sterilized, and especially, in minor operations on mouth, throat and teeth, the absorbent cotton, instead of being thrown on the floor should be immersed in water, a receptacle near at hand for that purpose, as I had the pleasure of seeing a fellow-worker use a vessel for this purpose and thereby doing away with the usual unsightly cotton-catcher or thrown over the floor, laden with the diseases of the mouth, nose and throat.

It is said by an old writer and philosopher first impressions should be good, and if not, the second will not be likely to follow, and cleanliness is certainly next to godliness, and in any case, we can preach as good a gospel in our offices as the ministers in the pulpit and at least we can help towards that grand time when we shall eliminate sin, disease and death.

A Co-Worker,

Francis G. Crouch, D. D. S.

Atlanta National Bk. Bldg., Atlanta, Ga.

WILLIAM O. HITCHCOCK, M. D., Dallas, Ga.

Dallas, Georgia, May 10th, 1911.

Journal-Record of Medicine,
Atlanta, Georgia.

Dear Sir:—For 23 years I have been a subscriber of the Journal-Record of Medicine. I was graduated at the Southern Medical College, March the 2nd, 1889. I am 49 years of age. I think the Journal-Record of Medicine is one of the best, cleanest and most instructive of any small Journal that I have seen or taken. I am anxious and ready for it every month.

Long live the editors and manager of the Journal--Record of Medicine!

Yours fraternally,
**WILLIAM O. HITCHCOCK, M.D.**

## Figures and Tables

**Figure f1:**